# G-rich DNA-induced stress response blocks type-I-IFN but not CXCL10 secretion in monocytes

**DOI:** 10.1038/srep38405

**Published:** 2016-12-12

**Authors:** Anna-Maria Herzner, Steven Wolter, Thomas Zillinger, Saskia Schmitz, Winfried Barchet, Gunther Hartmann, Eva Bartok, Martin Schlee

**Affiliations:** 1 Institute of Clinical Chemistry and Clinical Pharmacology, University Hospital Bonn, Bonn, Germany; 2 German Center for Infection Research (DZIF), Cologne-Bonn, Germany

## Abstract

Excessive inflammation can cause damage to host cells and tissues. Thus, the secretion of inflammatory cytokines is tightly regulated at transcriptional, post-transcriptional and post-translational levels and influenced by cellular stress responses, such as endoplasmic reticulum (ER) stress or apoptosis. Here, we describe a novel type of post-transcriptional regulation of the type-I-IFN response that was induced in monocytes by cytosolic transfection of a short immunomodulatory DNA (imDNA), a G-tetrad forming CpG-free derivative of the TLR9 agonist ODN2216. When co-transfected with cytosolic nucleic acid stimuli (DNA or 3P-dsRNA), imDNA induced caspase-3 activation, translational shutdown and upregulation of stress-induced genes. This stress response inhibited the type-I-IFN induction at the translational level. By contrast, the induction of most type-I-IFN-associated chemokines, including Chemokine (C-X-C Motif) Ligand (CXCL)10 was not affected, suggesting a differential translational regulation of chemokines and type-I-IFN. Pan-caspase inhibitors could restore IFN-β secretion, yet, strikingly, caspase inhibition did not restore global translation but instead induced a compensatory increase in the transcription of IFN-β but not CXCL10. Altogether, our data provide evidence for a differential regulation of cytokine release at both transcriptional and post-transcriptional levels which suppresses type-I-IFN induction yet allows for CXCL10 secretion during imDNA-induced cellular stress.

Inflammation is a vital physiological process that is essential for the detection and clearing of infections. Inflammatory cytokines are important mediators of this process, influencing cellular, local and global physiological functions, such as mRNA translation, immune cell infiltration, tissue perfusion and fever. Although these functions are essential for clearing the host of pathogens, they can be detrimental if excessively activated, as during septic shock, or constitutively active, as in autoinflammatory diseases. Thus, cytokine secretion is tightly regulated on the transcriptional level, and several important, pyrogenic cytokines, including Tumor Necrosis Factor α (TNF-α), Interleukin (IL)-6 or IL-1β, are known to have additional layers of regulation at the post-transcriptional level^1^.

The specific post-transcriptional regulatory mechanisms affecting the secretion of inflammatory cytokines are often determined by structures or sequences in the 3′UTR. Many cytokines, such as TNF-α and IL-1β have AU-rich elements (ARE) in their 3′UTR that are targeted by ARE-binding proteins which influence translation or transcript stability^1^,^2^,^3^,^4^. In addition, targeting of cytokine transcripts by miRNAs or lncRNAs has been described^1^.

More global post-transcriptional regulatory mechanisms that may influence an inflammatory response target the translation initiation machinery. Major mechanisms which are well-studied include eIF2α phosphorylation and the regulation of eIF4E by phosphorylation or sequestration by hypophosphorylated 4E-Binding Proteins (4E-BPs)^1^,^5^. While eIF2α phosphorylation inhibits the introduction of the methionyl-tRNA into the initiation complex, hypophosphorylated 4E-BPs inhibit the association of the preinitiation complex with the 5′ cap of mRNA^5^. eIF2α is phosphorylated during the activation of the integrated stress response during endoplasmatic stress or by Protein Kinase RNA-Activate (PKR) activation during viral infection^6^. 4E-BP phosphorylation is dependent on an active Mammalian Target Of Rapamycin (mTOR) pathway, which can be inhibited by starvation or activated, for example, during LPS stimulation, contributing to enhanced translation of a subset of proinflammatory cytokines, including IL6, TNFα or Chemokine (C-X-C Motif) Ligand 1 (CXCL1)^7^. In addition to these phosphorylation-dependent initiation regulatory mechanisms, apoptotic caspases have been shown to target translation initiation factors, such as eIF4G or eIF2, for cleavage during programmed cell death as a further mechanism of global translational control^8^.

A specialized form of inflammation, the type-I-IFN response, is engaged during viral infection. Since viral proliferation relies on the host cells and viruses thus consist of common cellular material, they lack the characteristically foreign structures found on many cellular microbes, such as bacterial or yeast cell wall components. Thus, viral detection relies on the recognition of foreign nucleic acids in the endosome or cytosol^9^. In the endosome, Toll-Like Receptors (TLRs) recognize double-stranded (TLR3), single-stranded RNA (TLR7, 8), or DNA containing unmethylated CpG motifs (TLR9)^10^,^11^,^12^,^13^,^14^,^15^. In the cytosol, the Retinoic Acid Inducible Gene-I (RIG-I)-like receptors (RLR) RIG-I and Melanoma Differentiation-Associated Protein-5 (MDA-5) recognize triphosphorylated, double-stranded RNA (3P-dsRNA) or polyinosic-polycytidylic acid (pI:C) as well as less defined motifs in highly structured RNA, respectively^16^,^17^,^18^. Cytosolic DNA triggers activation of cyclic-GMP-AMP (cGAMP) synthase (cGAS), a ligand-activated enzyme that produces the second messenger cGAMP, which in turn activates Stimulator of Interferon Genes (STING)^19^,^20^,^21^,^22^,^23^,^24^,^25^,^26^. cGAS and RLR engage IRF3 and/or 7 and other transcription factors such as Nuclear Factor Kappa-light-chain-enhancer of Activated B Cells (NFκB) and Activator Protein 1 (AP-1) to induce the transcription of type-I-IFNs as well as a range of chemokines and cytokines^9^. Type-I-IFNs are essential for anti-viral defence, as they induce anti-viral proteins in an autocrine and paracrine manner and direct and modulate the anti-viral immune response^27^. Furthermore, type-I IFN release is typically accompanied by inflammatory chemokines which also have have many essential and non-redundant roles in the anti-viral defence. Of particular importance is the C-X-C motif chemokine 10 (CXCL10), which has been shown to induce chemotaxis of macrophages, dendritic cells, NK cell and activated T lymphocytes to inflamed, infected or neoplastic entities^28^ and can also be induced independently of type-I-IFN^29^. CXCL10 has protective functions in a range of viral infections and can inhibit tumor growth via its angiostatic activity^28^,^30^.

Cellular stress responses can induce and modulate the type-I-IFN response. PKR activation by dsRNA, for example, has been shown to lead to a global translational shutdown, yet it allows for the translation of cytokine mRNAs such as IFN-β and IL6^31^. In addition, apoptosis has been shown to be able to induce a type-I-IFN response intrinsically which is counteracted by the apoptotic caspases to ensure the non-inflammatory nature of this “silent” cell death^32^,^33^. Nonetheless, the mechanisms of these modulations remain poorly understood, and post-transcriptional regulation of type-I-IFN and associated chemokines and cytokines during stress responses has not been studied extensively to date.

Here, we describe a novel form of post-transcriptional modulation of the type-I-IFN response that was inducible via cytosolic delivery of ODN2216-like G-rich immunomodulatory DNA (imDNA) to human monocytes. Independent of TLR9, the primary target of ODN2216, type-I-IFN secretion induced by cytosolic nucleic acid stimuli was downregulated by a global blockade of translation while CXCL10 escaped this modulation concurrent with enhanced translation. Post-transcriptional modulation was independent of classical translation control mechanisms and associated with the activation of caspase-3 and the upregulation of the stress-associated genes ATF4 and CHOP. Consistently, imDNA co-transfection could be partially simulated by direct activation of mitochondrial apoptosis using the bcl-2-inhibitor ABT737. Although pan-caspase-inhibitors could not fully countermand translational blockage by imDNA, they could rescue IFN-β secretion by a dramatic increase in transcript levels both in imDNA and ABT737-treated cells, indicating regulation of both IFN-β transcript levels and translation induced by imDNA-induced cell stress during a type-I-IFN response. Under the same conditions, CXCL10 transcript levels, translation and secretion remained largely unaffected by imDNA co-transfection, ABT737 treatment or caspase inhibition, indicating the presence of regulatory mechanisms which guarantee the maintenance of chemokine secretion under these stress conditions.

## Results

### ODN2216-like DNA blocks monocytic IFN-α secretion induced by cytosolic nucleic acid stimuli

**Figure 1:**
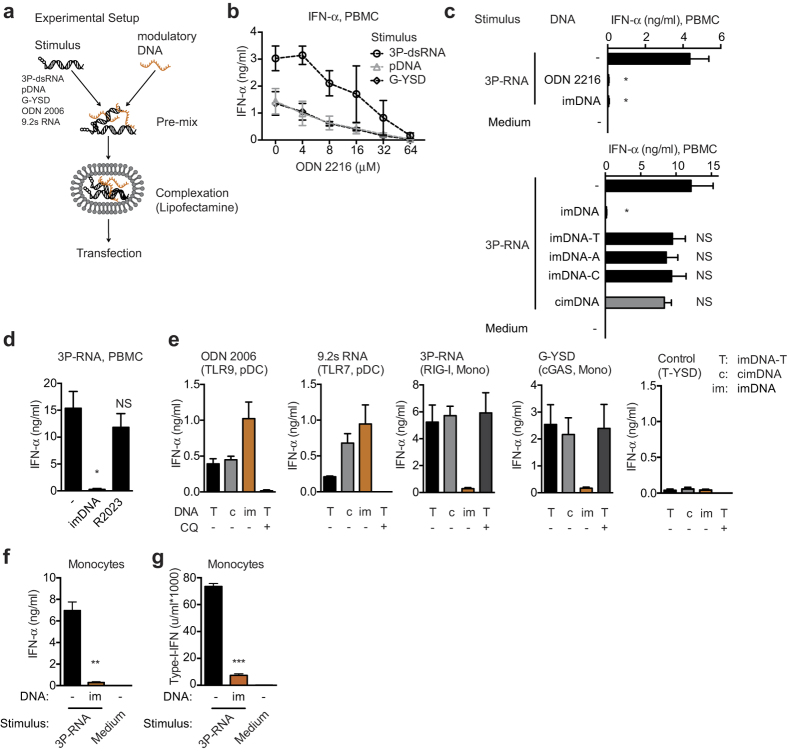
imDNA inhibits monocytic IFN-α secretion induced by cytosolic stimuli. (**a**) Protocol applied throughout the study: Interferon-inducing nucleic acids are mixed in transfection medium with modulatory DNA, then mixed with Lipofectamine 2000 for complexation and added to cells for their transfection. (**b**–**e**) IFN-α concentrations in the supernatants of PBMC, treated with chloroquine (2.5 μg/ml), if not indicated otherwise, and transfected with indicated stimuli/ODN. IFN-α concentrations in the supernatants were measured 20 h post transfection. (**b**) Dose-titration (4–64 μM ≈ 12.5–100 ng/96-well) of ODN2216 with consistent quantities (100 ng) of cytosolic stimuli. Nucleic acid quantities were stocked up with the inert ODN2216 (G → T) (see Table 1) to a total of 200 ng/well. (**c**,**d**) Co-transfection of 3P-RNA and DNA or RNA oligomers as indicated. (**e**) Co-transfection of nucleic acid stimuli (ODN 2006, 9.2 s RNA, 3P-RNA, G-YSD or T-YSD as negative control) and modulating DNAs (imDNA-T (T; black and dark grey), cimDNA (c; light grey), imDNA (im; vermilion)), with or without chloroquine pretreatment (CQ). pDC: Plasmacytoid dendritic cells; Mono: Monocytes (**f**,**g**) Monocytes were CD14^+^ MACSorted and (co)transfected with the indicated nucleic acids. IFN-α concentration (**f**) or type-I-IFN activity (**g**) was detected 20 h after transfection. (**c**,**d**,**f**): NS, not significant (P > 0.05); *P ≤ 0.95; *P ≤ 0.01; ***P ≤ 0.001 (repeated measures one-way analysis of variance (ANOVA) followed by Fisher’s LSD post-hoc test). (**b**–**f**) Data are pooled from two experiments with one or two biological replicates in each experiment (mean and s.e.m. of n = 3 donors (**b**,**e**) or n = 4 donors (**c**,**d**,**f**,**g**)).

In order to investigate the crosstalk between different type-I-IFN inducing nucleic acid recognition pathways, we transfected peripheral blood mononuclear cells (PBMC) with combinations of a range of immunostimulatory nucleic acids. Unexpectedly, we observed that ODN2216 reproducibly and dose-dependently inhibited the type-I-IFN secretion induced by cytosolic immunostimulatory nucleic acids (triphosphorylated double-stranded RNA (3P-dsRNA), plasmid DNA and G-rich Y-form short DNA (G-YSD^34^) in chloroquine-treated PBMC (Fig. 1a,b). ODN2216 as an A-type TLR9 ligand has been designed to induce type-I-IFN secretion in plasmacytoid dendritic cells (pDC) without complexation^35^. However, chloroquine inhibits endosomal acidification, a prerequisite for the activation of the endosomal TLRs (TLR3, 7 and 9), strongly suggesting that the observed effect of ODN2216 under these conditions was TLR9-independent. Furthermore, the main producers of type-I-IFNs in the peripheral blood after chloroquine treatment are monocytes^17^,^34^,^36^, which do not express TLR9.

ODN2216 is a 20 mer that is defined by a central 10 nt palindrome containing three unmethylated cytidine-phosphate-guanosine (CpG) motifs and 5–6 consecutive deoxyguanosines (dG) at each end. While the unmethylated CpG motifs are the recognition motif for TLR9, the dG ends as well as its unmodified DNA backbone define ODN2216 as an A-type CpG oligodeoxynucleotide (ODN). The dG ends form G-tetrads, a G-specific non-Watson-Crick conformation composed of four dG in a planar arrangement featuring very high melting temperatures and enabling the formation of intermolecular high-molecular-weight agglomerates that can be phagocytised by pDCs to activate TLR9^35^.

To further investigate whether the inhibitory activity of ODN2216 was indeed independent of TLR9 recognition, we specifically disrupted the CpG motives within the central palindrome of ODN2216, which did not affect its inhibitory activity on 3P-dsRNA induced IFN-α secretion (Fig. 1c, upper graph, for sequences, see Table 1). Taken together with the observation that chloroquine cannot block its inhibitory action, we concluded that TLR9 was dispensable for the inhibitory action of ODN2216. However, to exclude the interference of the TLR9 pathway with further experiments, we decided to use the CpG-switched ODN (imDNA) as our model DNA.

To study the role of the terminal guanosines in imDNA, we exchanged them for cytidines (imDNA-C), adenosines (imDNA-A) or thymidines (imDNA-T), and thereby restored IFN-α secretion upon co-transfection with 3P-dsRNA, indicating that terminal guanosines are essential for the inhibitory activity (Fig. 1c, lower graph). To evaluate the role of the central palindromic sequence for the inhibitory activity, we tested a range of mutations but did not obtain conclusive results. However, among those mutants, one ODN comprised a single central A → T mutation reproducibly leading to the loss of its inhibitory activity across several batches (control-imDNA; “cimDNA”, Fig. 1c, lower graph), demonstrating influence of the central non-dG sequence on the activity of imDNA. In addition, to investigate the contribution of the deoxyribose-phosphate backbone, we co-transfected an RNA-oligonucleotide (ORN) with the same structural features as imDNA. However, co-transfection of ORN did not affect IFN-α secretion (Fig. 1d, R2023), indicating that the observed inhibitory imDNA-effect depended on the DNA backbone and is not simply caused by cytosolic G-tetrad induced nucleic acid agglomerates.

To characterize the cell-type specificity of imDNA inhibitory activity, we transfected PBMC with specific nucleic acid stimuli that activate nucleic acid sensors that are differentially expressed by monocytes or pDCs: in pDCs, TLR7 (9.2sRNA) and TLR9 (ODN 2006); in monocytes, RIG-I (3P-RNA) and cGAS (G-YSD)^17^,^34^,^35^,^37^,^38^. T-YSD transfection served as a non-stimulatory control (Fig. 1e)^34^. While imDNA but not cimDNA or imDNA-T efficiently blocked IFN-α secretion induced by the cytosolic, monocyte activating stimuli 3P-dsRNA and G-YSD, TLR-dependent IFN-α secretion by pDCs was not inhibited but rather enhanced (Fig. 1e). On the other hand, chloroquine pre-treatment efficiently blocked TLR/pDC activation but not cytosolic/monocyte stimulation (Fig. 1e). To exclude crosstalk between different cell types in the PBMC population, we isolated CD14^+^ monocytes from PBMCs, co-transfected these and observed inhibition of 3P-dsRNA-induced IFN-α secretion upon imDNA co-transfection, comparable to the results obtained by PBMC co-transfection (Fig. 1f).

Furthermore, we measured the type-I-IFN activity in the supernatant with HEK-Blue IFN-α/β reporter cells, which is a functional assay detecting bioactive type-I IFN, and observed drastically reduced activity in supernatants of 3P-RNA:imDNA cotransfected cells compared to 3P-RNA transfected cells (Fig. 1g).

To exclude imDNA-mediated effects on the delivery of the stimulus, we transfected PBMC with separate complexations of imDNA and 3P-RNA (Supplementary Fig. S1), which led to a reduced but still significant inhibition of IFN-α secretion, excluding simple blockage of delivery as an inhibitory mechanism.

In conclusion, our data indicate that monocytic secretion of IFN-α after cytosolic nucleic acid stimulation can be robustly and cell-intrinsically inhibited by short DNA comprising G-rich ends.

### Post-transcriptional modulation of cytokine secretion by imDNA

**Figure 2:**
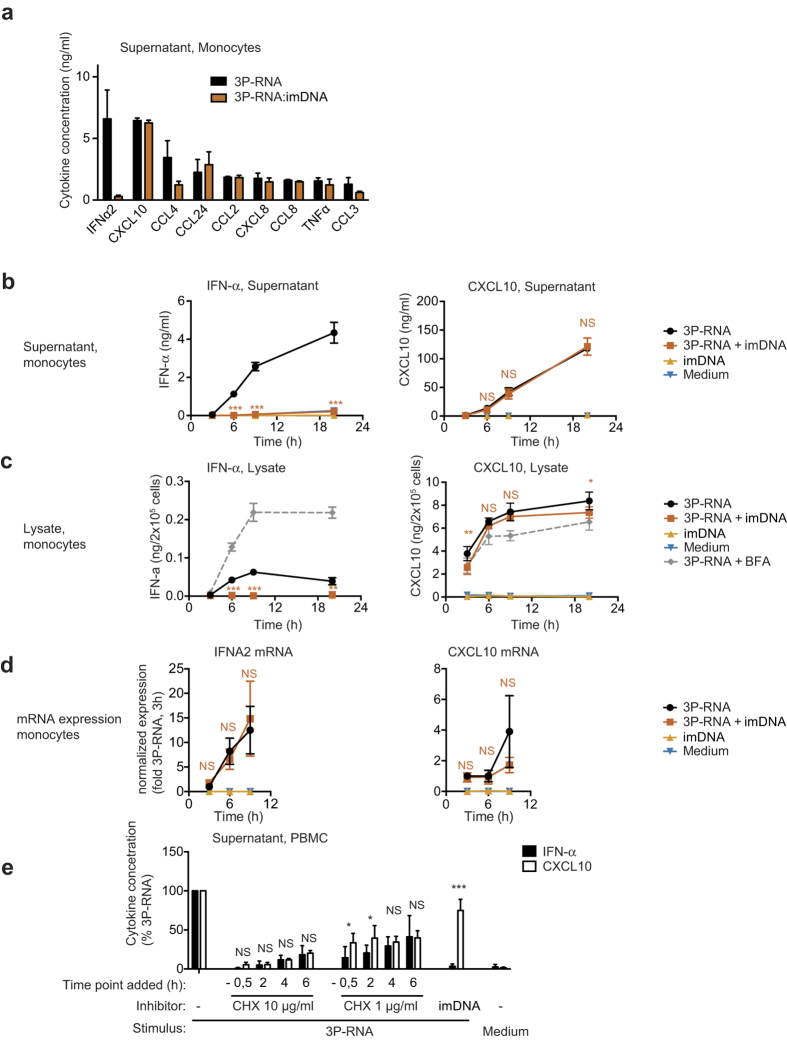
Differential post-transcriptional regulation of cytokine secretion by imDNA co-transfection. (**a**) Concentrations of the 8 most highly concentrated cytokines in the supernatant of 3P-RNA transfected (black) or 3P-RNA:imDNA (vermilion) co-transfected monocytes, 20 h post transfection. (**b**,**c**) IFN-α (left) or CXCL10 (right) concentrations in the supernatants (**b**) or lysates (**b**) of monocytes, transfected with 3P-RNA and/or imDNA, measured at the indicated time points after transfection. (**c**) BFA: Cells were treated with brefeldin A (1 μg/ml) (**d**) mRNA expression of IFNA2 (coding for IFN-α2, left) or CXCL10 (coding for CXCL10, right), normalized to ACTB mRNA, measured by quantitative RT-PCR at the indicated time points. Results are displayed as relative to those measured for 3P-RNA transfected cells (3 h), set as 1 (**e**) IFN-α (black) or CXCL10 (white) concentrations in the supernatants of chloroquine-treated PBMC 20 h post transfection. The cells were transfected with 3P-RNA or 3P-RNA and imDNA and treated with 10 μg/ml or 1 μg/ml cycloheximide (CHX) at the indicated time points (relative to 3P-RNA transfection time point). Data are displayed as relative to those of 3P-RNA transfected cells, set as 100%. (**b**–**e**) NS, not significant (P > 0.05); *P ≤ 0.05; **P ≤ 0.01; ***P ≤ 0.001 (repeated measures two-way ANOVA followed by Bonferroni’s post-hoc test). (**b**–**d**) P-values indicated only for comparison of 3P-RNA transfection and 3P-RNA:imDNA co-transfection. (**e**) Statistical testing compares the relative concentrations of IFN-α and CXCL10. (**a**–**f**) Data are pooled from two experiments with one or two biological replicates in each experiment (mean and s.e.m. of n = 3 donors (**a**) or n = 4 donors (**b**–**e**)).

To investigate whether the inhibitory activity of imDNA affects other 3P-dsRNA-induced cytokines, we subjected monocytic supernatants to multi-cytokine analysis. Again, we could detect pronounced differential regulation of IFN-α of 3P-dsRNA transfected vs. 3P-dsRNA:imDNA co-transfected monocytes (Fig. 2a). However, among the most highly secreted chemokines and cytokines, only Chemokine (C-C Motif) Ligand (CCL)3 and CCL4 showed mild suppression of 3P-dsRNA-induced secretion upon imDNA co-transfection. By contrast, other highly secreted cytokines and chemokines, such as CXCL10, CCL24 or CCL2, showed no differential regulation.

To determine the kinetics of imDNA inhibition, we performed a time course using monocytes highly enriched by CD14^+^ magnetic bead-assisted cell sorting. We could observe that reduction of 3P-dsRNA-induced IFN-α secretion by co-transfection of imDNA was detectable as early as 3 h after transfection, reaching significance at 6 h after stimulation (Fig. 2b, left graph). However, when we measured CXCL10, we could not detect any imDNA-dependent decrease in 3P-dsRNA-induced secretion at any time point that we tested (Fig. 2b, right graph). To approximate the source of this differential regulation, we first tested whether IFN-α was retained within the cells by detection of cytokine concentration in cellular Triton-X-100 lysates. While blockade of protein secretion as a positive control using brefeldin A 2 h after 3P-dsRNA-stimulation led to the accumulation of IFN-α within cell lysates^39^, no IFN-α was detectable in lysates of 3P-dsRNA:imDNA co-transfected cells. Nonetheless, intracellular CXCL10 levels remained largely unaffected with a significant, but only very mild reduction at 3 h and 20 h post transfection (Fig. 2c). Next, we measured mRNA levels in 3P-dsRNA-transfected and 3P-dsRNA:imDNA-co-transfected cells and, in contrast to our observations for IFN-α protein, could not detect any reduction in IFNA2 transcript levels in 3P-dsRNA:imDNA co-transfected cells, suggesting largely unaffected 3P-dsRNA-induced IFNA2 transcription concomitant with post-transcriptional regulation upon imDNA co-transfection (Fig. 2d).

The differential secretion of IFN-α and CXCL10 observed at all time points was a strong indication of differential translational regulation. However, differences in secretion kinetics could account for the observed differences in end-point concentrations. To better understand the kinetics of IFN-α and CXCL10 release, we simulated a global translational blockade at different time points by addition of cycloheximide during 3P-dsRNA stimulation. We added cycloheximide in two different concentrations (1 μM and 10 μM), 0.5 h before or 2, 4, or 6 h after stimulation with 3P-dsRNA (Fig. 2e). In general, CXCL10 secretion was inhibited by cycloheximide to an extent comparable to IFN-α secretion. We only observed significantly stronger inhibition of IFN-α secretion than of CXCL10 secretion at 1 μM CHX at the two earliest timepoints, −0.5 h and 2 h. In contrast, differential secretion was much more pronounced for imDNA co-transfection (Fig. 2e), indicating that effects of secretion dynamics could not sufficiently explain differential end-point cytokine concentrations.

To test whether imDNA mediated post-transcriptional regulation affects only transfected cytosolic stimuli, we applied uncomplexed 2′3′cGAMP directly to monocytes and simultaneously transfected them with imDNA or imDNA-T (Supplementary Fig. S2)^40^. Here, we could again observe suppression of IFN-α secretion but not transcription by imDNA but not imDNA-T, while CXCL10 induction was mildly suppressed on both the mRNA as well as the protein level. These data confirm that the differential translational regulation of IFN-α and CXCL10 secretion can also occur for non-transfected cytosolic stimuli.

Altogether, we conclude that imDNA co-transfection leads to differential regulation of IFN-α but not CXCL10 secretion at the post-transcriptional level.

### Differential translation of IFN-β and CXCL10 during an imDNA-induced stress response

**Figure 3:**
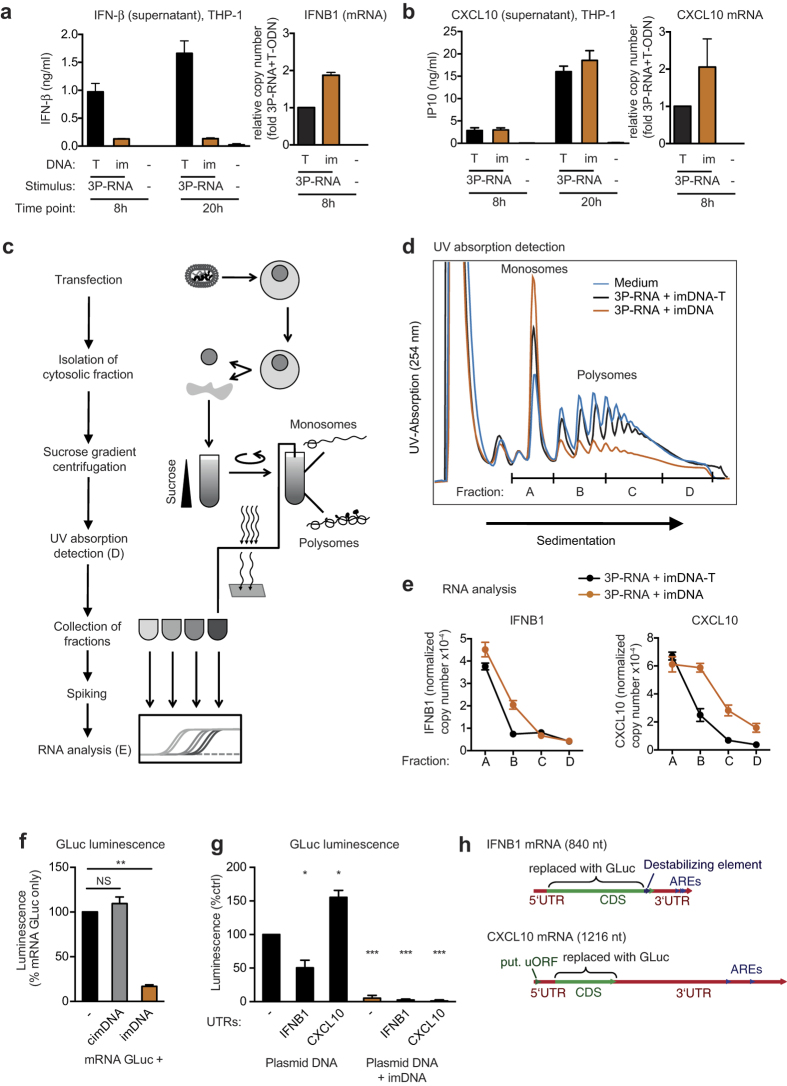
Differential translation of IFNB1 and CXCL10 during imDNA co-transfection. (**a**,**b**) IFN-β (**a**) or CXCL10 (**b**) concentrations in the supernatant (left) or IFNB1 (**a**) or CXCL10 (**b**) mRNA expression of THP-1 transfected with 3P-RNA (3P-RNA) and imDNA-T (black, “T”) or imDNA (vermilion, “im”), 8 h or 20 h post transfection. mRNA expression was normalized to GAPDH and results are displayed as relative to those of 3P-RNA:imDNA-T co-transfection, set as 1. (**c**) Flow chart of polysome separation by sucrose gradient ultracentrifugation. THP-1 were transfected with 3P-RNA and imDNA-T or imDNA. 8 h later, cells were lysed and the cytosolic fraction loaded onto a 10–50% sucrose gradient. After ultracentrifugation, the ribonucleic particles were detected by UV absorption (results in **d**). Afterwards, collected fractions were divided into four fractions. Fractions were spiked with artificial GLuc mRNA, purified and analysed by qPCR (results in **e**). (**d**) UV absorption of the ribonucleoparticles after gradient ultracentrifugation. Displayed are the UV absorption profiles of THP-1, treated with medium (blue), 3P-RNA:imDNA-T co-transfection (black) or 3P-RNA:imDNA co-transfection (vermilion). Fractions analysed in (**e**) are indicated below. (**e**) IFNB1 (left) and CXCL10 (right) mRNA, detected in the respective fractions of THP-1, transfected with 3P-RNA and imDNA-T (black) or imDNA, respectively (vermilion). Data are normalized to spiked-in GLuc mRNA. (**f**,**g**) GLuc activity in the supernatant of THP-1, 20 h after transfection of an artificial GLuc mRNA (**f**) or plasmid constructs (**g**) with or without cimDNA or imDNA. (**g**) Plasmids encode eF1α promoter-driven GLuc, flanked with regulatory sequences, encompassing annotated 5′ and 3′ UTR of CXCL10 (see **h**) or annotated 5′ and 3′ UTR as well as a destabilizing sequence within the CDS of IFNB1 (here not translated, see **h**). (**h**) IFNB1 and CXCL10 mRNA structures indicating GLuc insertion. ARE (AU-rich element), uORF (upstream open reading frame): Putative regulatory sequences (**a**,**b**,**d**–**g**) Data are pooled from two (**a**) or three (**b**,**f**,**g**) biological replicates (mean and s.e.m.) or representative of three independent experiments (**d**,**e**) and displayed as technical duplicates (**e**; mean and s.d.). (**f**,**g**) NS, not significant (P > 0.05); *P ≤ 0.05; **P ≤ 0.01; ***P ≤ 0.001 (repeated measures one-way ANOVA followed by Fisher’s LSD post-hoc test).

To investigate the translation of CXCL10 and type-I-IFN during imDNA co-transfection, we performed polysome separation by sucrose gradient centrifugation with THP-1 monocytes. Since THP-1 do not express IFN-α^41^, we measured IFN-β and CXCL10, 8 h and 20 h after transfection of 3P-dsRNA, together with either imDNA or imDNA-T. Here, we observed the same expression pattern as for IFN-α and CXCL10 in primary monocytes: IFN-β secretion was suppressed upon imDNA co-transfection while IFN-β transcript levels as well as CXCL10 transcript levels and secretion remained unaffected or demonstrated a mild increase (Fig. 3a,b). For polysome separation, we stimulated THP-1 for 8 h, isolated the cytosolic fraction, subjected this fraction to sucrose gradient centrifugation and detected ribonuclear complexes by absorption at 254 nm (Fig. 3c). While 3P-dsRNA:imDNA-T co-transfection did not significantly alter the composition of the ribosomal complexes compared to medium-treated cells, we observed substantial suppression of polysome formation in 3P-dsRNA:imDNA co-transfected THP-1 (Fig. 3d) demonstrating a global translational inhibition induced by imDNA transfection. This global translational blockade was accompanied by the induction of classical markers of stress responses, Activating Transcription Factor 4 (ATF4) and C/EBP-Homologous Protein (CHOP), indicating a profound stress response induced by imDNA (Supplementary Fig. S3a,b)^6^. To test for differential translation of IFN-β and CXCL10, we isolated RNA from four fractions containing ribosomal complexes of different orders (indicated in Fig. 3c,d). In these fractions, we could detect a shift of ATF4 and CHOP transcripts towards higher complexes in 3P-dsRNA:imDNA-treated cells when compared to 3P-dsRNA:imDNA-T treated cells indicating an increased translation of ATF4 and CHOP, which is a further hallmark of the activation of the integrated stress response (Supplementary Fig. S3c,d)^6^. Similarly, CXCL10 mRNA was shifted towards higher order ribonuclear particles despite global translation inhibition (Fig. 3e, right graph). By contrast, IFNB1 mRNA shifted towards monosomes and oligosomes of lower order, indicating a translational suppression (Fig. 3e, left graph). Our data indicate that imDNA induces a global inhibition of translation leading to the suppression of IFN-β translation, while CXCL10 demonstrates enhanced translation despite global translational suppression.

Next, to test whether the inhibitory activity of imDNA was specific for IFNB1, we co-transfected an artificial mRNA coding for the reporter Gaussia Luciferase (GLuc), generated by *in-vitro* transcription, with cimDNA or imDNA and observed profound inhibition of translation even of this artificial mRNA (Fig. 3f). Furthermore, we attempted to generate artificial mRNAs encoding GLuc, flanked by the annotated 5′ and 3′ UTRs of CXCL10 and IFNB1 mRNA (NCBI refseq NM_002176 and NM_001565, respectively); however, their quality varied to a degree that made comparison impossible. To investigate the effect of UTR sequence on translation inhibition, we directly transfected plasmids with eF1α-promoter-driven GLuc, unflanked or flanked with CXCL10 UTRs or IFNB1 UTRs including a short sequence of the IFNB1 CDS that had been described to modulate IFNB1 mRNA stability (indicated in Fig. 3h)^42^. Here, 20 h after transfection, mildly, but significantly, reduced GLuc activity could be detected for IFNB1-UTR-flanks while CXCL10 UTR flanks increased the GLuc activity, suggesting regulation by the respective UTRs under normal IFN-inducing conditions, activated by the transfected plasmid DNA (Fig. 3g). When we co-transfected imDNA with the plasmids, however, GLuc activity was almost completely abrogated for all three constructs (Fig. 3g). Thus, this experimental set-up did not reflect differential translational regulation of the endogenous IFNB1 and CXCL10 mRNAs. We therefore concluded that either plasmid transfection itself is too toxic in combination with imDNA to reflect 3P-dsRNA:imDNA co-transfection or the differential regulation of CXCL10 and IFN-β translation is not due to UTR-encoded regulatory motifs.

Our data demonstrate that imDNA induces a global inhibition of translation leading to suppression of IFN-β translation that also affects synthetic mRNAs, while CXCL10 escapes this inhibition by enhanced translation by mechanisms possibly not encoded in the respective UTRs.

### imDNA activity is independent of innate cytosolic DNA receptors and DNA damage signalling

**Figure 4:**
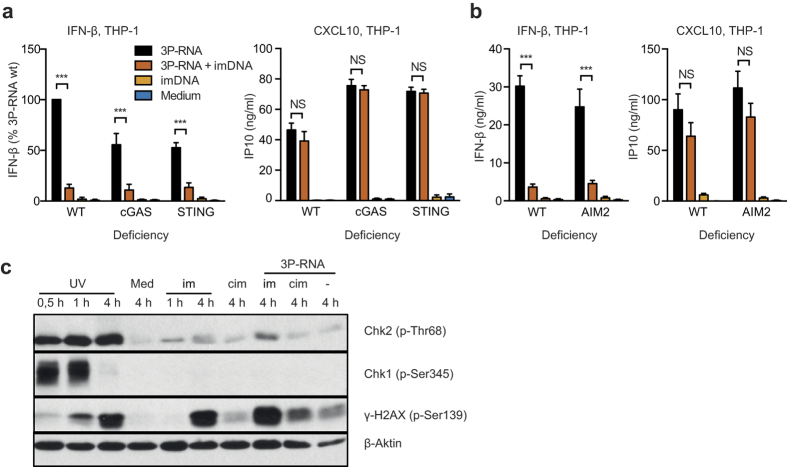
imDNA activity is independent of innate cytosolic DNA receptors and DNA damage signaling. (**a**,**b**) IFN-β (left) or CXCL10 (right) concentrations in the supernatants of wild-type THP-1 or THP-1, deficient in the indicated proteins, transfected with 3P-RNA and/or imDNA (3P-RNA: black; 3P-RNA:imDNA: vermilion; imDNA: orange; medium control: blue). Left: Results are presented as relative to those of wild-type cells, transfected with 3P-RNA alone. Data are pooled from three (**a**) or six (**b**) independent experiments. NS, not significant (P > 0.05); ***P ≤ 0.001 (repeated measures two-way analysis of variance (ANOVA) followed by Bonferroni’s post-hoc test). (**c**) Immunoblot analysis of the indicated phospho-proteins of total cell lysates at the indicated time points post UV irradiation or transfection with 3P-RNA and/or imDNA (im) or cimDNA (cim), respectively. Results are representative of three experiments.

Since imDNA activity depended on a DNA backbone and it shares some features of the G-YSD motif (G-rich ends, central sequence capable of forming a duplex ref. 34), we were interested whether either the imDNA-dependent translational suppression or the increased CXCL10 translation depended on the classical cytosolic DNA recognition pathways. Therefore, we co-transfected THP-1 clones genetically deficient in cGAS, STING or Absent In Melanoma-2 (AIM2) with 3P-dsRNA:imDNA complexes (Fig. 4a,b). However, none of these deficiencies rescued IFN-β secretion or significantly reduced CXCL10 secretion relative to the control, 3P-dsRNA transfection. Another pathway that can detect DNA and induce a stress response that could lead to translational shutdown is the DNA damage response^43^,^44^. Therefore, we tested whether the checkpoint kinases CHK1 or CHK2 or histone H2AX were phosphorylated upon imDNA transfection, classical markers for the activation of DNA damage signaling (Fig. 4c)^45^. CHK1 and CHK2 are phosphorylated by the DNA-damage response kinases Ataxia Telangiectasia Mutated (ATM) and Ataxia Telangiectasia And Rad3-Related Protein (ATR)^45^, while histone H2AX is phosphorylated at the site of DNA damage by the ATM, ATR and Protein Kinase, DNA-Activated (DNA-PK)^46^.

While the positive control, ultraviolet (UV) irradiation, led to a robust phosphorylation of CHK1, CHK2 and H2AX, we detected only weak phosphorylation of CHK2 und no detectable phosphorylation of CHK1 in imDNA-transfected cells (Fig. 4c). Furthermore, the inhibitory activity of imDNA remained unaffected by ATM/ATR/DNA-PK inhibitors Ku55933, CGK733 and Nu7062 (Supplementary Fig. S4). We therefore concluded that classical DNA damage signalling is not activated. By contrast, we detected phosphorylated (γ-)H2AX in imDNA-transfected THP-1 (Fig. 4b). However, γ-H2AX has also been shown to be activated during apoptosis-dependent DNA degradation (discussed below)^47^.

Thus, we could exclude classical DNA recognition mediated by cGAS, AIM2 as well as DNA damage sensors as the source for imDNA-induced translational shutdown.

### Modulation of IFN-β secretion by apoptotic caspases during imDNA co-transfection

**Figure 5:**
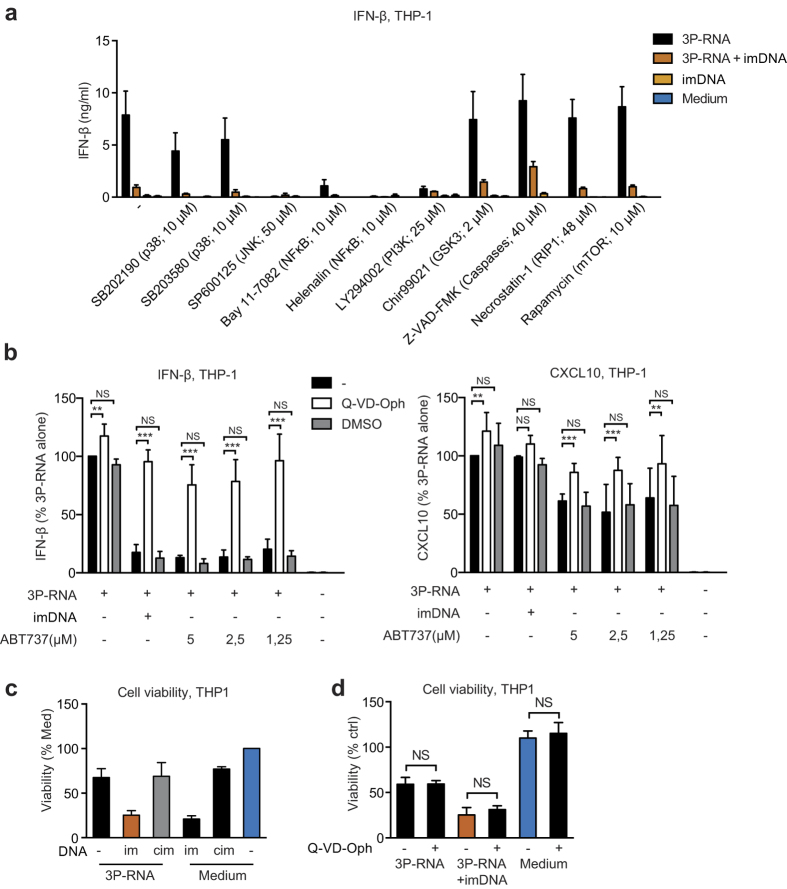
Pan-Caspase inhibitors restore imDNA- and ABT737-induced suppression of IFN-β secretion. (**a**) IFN-β in the supernatant of THP-1, 20 h post transfection with 3P-RNA and/or imDNA as indicated. Cells were pretreated for 1 h with the indicated inhibitors. Inhibitor targets are in brackets. (**b**) IFN-β (left) or CXCL10 (right) in the supernatant of THP-1, 20 h post transfection with 3P-RNA and/or imDNA. Cells were pretreated without inhibitor (black bars), with Q-VD-Oph (10 μM; white bars) or DMSO (1:2000; grey bars) 1 h before transfection. ABT737 was added 1 h post transfection to the indicated final concentrations. Results are presented as relative to those of untreated cells transfected with 3P-RNA alone, set as 100%. (**c**,**d**) Cell viability measured by metabolic activity on MTT, 20 h post transfection. Results are presented as relative to an untreated control. (**d**) im, imDNA; cim, cimDNA. NS, not significant (P > 0.05); **P ≤ 0.01; ***P ≤ 0.001 (repeated measures two-way analysis of variance (ANOVA) followed by Bonferroni’s post-hoc test). (**a**,**b**,**c**,**d**): Data are pooled from 3 (**a**,**b**,**c**) or two (**d**) experiments (mean and s.e.m.).

In order to determine which pathways are involved in imDNA-induced translational regulation, we employed a range of inhibitors targeting pathways involved in cell death or survival. Of these, inhibitors of JUN N-Terminal Kinase (JNK), NFκB and PI3-Kinase completely blocked IFN-β secretion by THP-1 (Fig. 5a) and were therefore excluded from further study. Inhibitors of p38 MAP-Kinase, Apoptosis Signal Regulating Kinase 1 (ASK-1), V-Akt Murine Thymoma Viral Oncogene (AKT), Glycogen Synthase Kinase 3 (GSK3), Receptor (TNFRSF)-Interacting Serine-Threonine Kinase 1 (RIPK1) or mTOR neither markedly influenced IFN-β secretion by 3P-dsRNA nor its suppression upon 3P-dsRNA:imDNA co-transfection. Only the pan-caspase inhibitor Z-VAD-FMK could restore IFN-β secretion upon 3P-dsRNA:imDNA co-transfection.

It has been previously observed that apoptotic caspases suppressed type-I-IFN induction during apoptosis, which is induced by DNA leaking from damaged mitochondria^32^,^33^. In these studies, a direct inducer of mitochondrial apoptosis, the B-Cell CLL/Lymphoma 2 (bcl-2) inhibitor ABT737, was employed to induce apoptosis and an optimized pan-caspase inhibitor, Q-VD-Oph, was used to inhibit caspase activation. The combination of both induced a type-I-IFN response, dependent on mitochondrial DNA recognition by the cGAS pathway^32^,^33^. Therefore, we tested whether apoptotic caspases activated by ABT737 could not only suppress type-I-IFN secretion induced by mitochondrial apoptosis but also induced by cytosolic nucleic acid stimulation. Indeed, when we applied ABT737 1 h after 3P-dsRNA transfection, IFN-β secretion was suppressed to a similar extent as upon 3P-dsRNA:imDNA co-transfection (Fig. 5b, left graph), and, as observed for 3P-dsRNA:imDNA co-transfection, CXCL10 secretion was only mildly reduced (Fig. 5b, right graph). The reduction in IFN-β secretion could be rescued by pre-incubation with Q-VD-Oph in both 3P-dsRNA:imDNA co-transfected THP-1 and 3P-dsRNA transfected, ABT737 treated THP-1, while Q-VD-Oph pre-incubation only mildly increased CXCL10 secretion (Fig. 5b).

The activating effects of the pan-caspase inhibitor raised the question whether cell death may also account for the effects observed during imDNA co-transfection. To examine imDNA-mediated cell toxicity, we measured cell survival 20 h after transfection using a 3-(4,5-dimethylthiazol-2-yl)-2,5-diphenyltetrazolium bromide (MTT) assay. Indeed, we could observe reduced metabolic activity in imDNA but not cimDNA cotransfected cells, which is indicative of cytotoxicity (Fig. 5c). However, quite unexpectedly, the pan-caspase inhibitor Q-VD-Oph had no effect on the viability of the cells, clearly indicating that there is a rescue mechanism for IFN-β secretion that is independent of cell viability (Fig. 5d).

**Figure 6:**
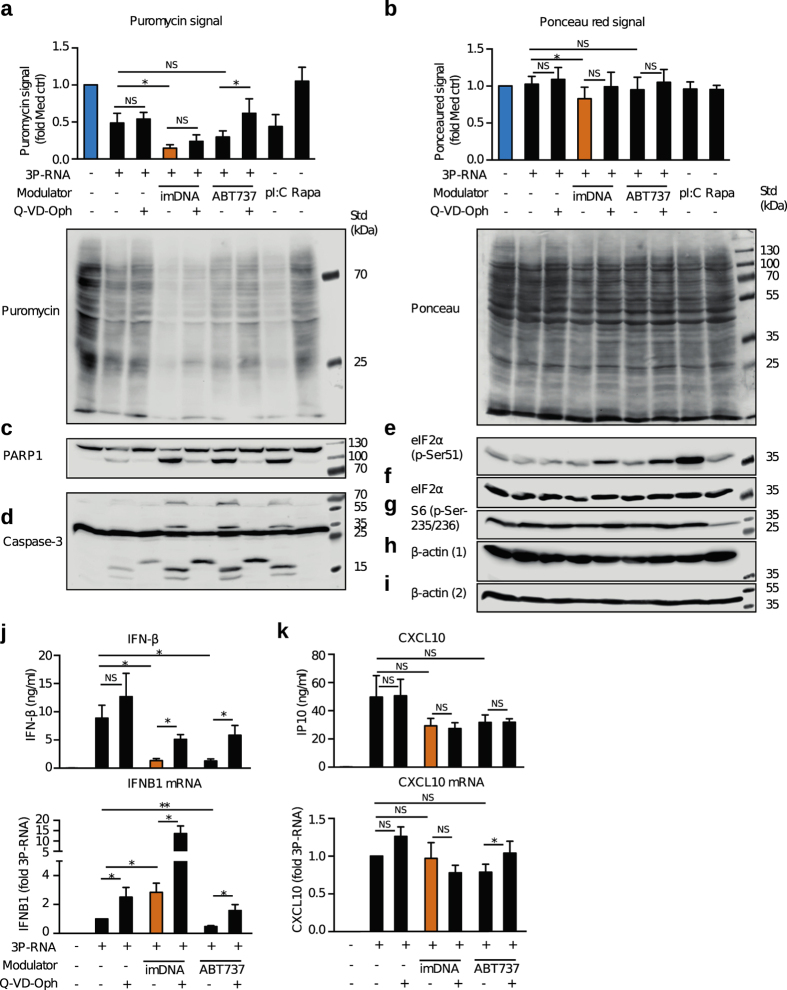
Pan-caspase inhibitor does not restore imDNA-suppressed translation but enhances IFNB1 transcription. (**a**) Immunoblot analysis of puromycin incorporated during 10 min incubation (1 μg/ml), 6 h post transfection of THP-1. Cells were pretreated for 1 h with Q-VD-Oph, transfected with 3P-RNA and/or imDNA or pI:C. ABT737 (5 μM) was added 1 h post transfection. Rapamycin (Rapa, 200 nM) was added simultaneously with transfection. Bottom: Image of one of three immunoblots. Top: Quantification of three immunoblots. For the quantification, the puromycin signal of nascent polypeptide chains, 55–100 kDa in length was analysed. Results are presented relative to those of untreated cells, set as 1. (**b**) Ponceau red signal of the immunoblot membranes analyzed in (**a**), analyzed and presented as in (**a**). (**c**–**i**) Immunoblot analyses of the indicated (phospho-)proteins in THP-1 treated as in (**a**). (**h**) Loading control for (**a**–**c**,**e**,**f**); (**i**) Loading control for (**d**,**g**). (**j**) IFN-β concentration in the supernatant, 20 h post transfection (top) or IFNB1 mRNA expression, 6 h post transfection, of THP-1, treated as in (**a**). (L) CXCL10 concentration in the supernatant 20 h post transfection (top) or CXCL10 mRNA expression, 6 h post transfection, of THP-1, treated as in (**a**). (**j**,**k**) Bottom: mRNA expression was normalized to GAPDH mRNA expression and results are presented as relative to those of cells transfected with 3P-RNA alone, set as 1. (**a**,**b**,**j**,**k**): NS, not significant (P > 0.05); *P ≤ 0.05; **P ≤ 0.01; (repeated measures one-way analysis of variance (ANOVA) followed by Fisher’s LSD post-hoc test). (**a**–**l**) Data are pooled from four (**a**,**b**) or three (**j**,**k**) (mean and s.e.m.), or representative of four (**a**,**b**, bottom) or three (**c**–**i**) experiments. All immunoblots displayed are from the same experiment.

To identify the origin of this Q-VD-Oph-induced increase in IFN-β secretion, we tested whether application of Q-VD-Oph rescued translational activity in 3P-dsRNA:imDNA co-transfected cells using the SUnSET Assay, which detects puromycin incorporation^48^. THP-1 cells were pre-treated with Q-VD-Oph and transfected with 3P-dsRNA or 3P-dsRNA:imDNA or transfected with 3P-dsRNA and treated with ABT737 1 h after transfection. As positive controls for translational control mechanisms, we also used pI:C transfection and rapamycin (discussed below). After 6 h, we treated the cells for 10 min with 1 μg/ml puromycin to allow its incorporation into peptide chains (SUnSET Assay)^48^. Then cells were harvested for qPCR and immunoblot analysis. Although puromycin incorporation was mildly suppressed by 3P-dsRNA transfection alone (Fig. 6a), 3P-dsRNA:imDNA co-transfected cells demonstrated a profound suppression in puromycin levels. Ponceau red staining was used as a loading control (Fig. 6b) and was significantly reduced only for 3P-dsRNA-imDNA co-transfection. However, this reduction was much less pronounced than in the SUnSET assay (Fig. 6a,b). In contrast to imDNA co-transfection, ABT737 addition only induced a mild translational repression in some experiments (Fig. 6a).

We also investigated caspase-3 activation in these lysates. Both imDNA co-transfection as well as ABT737 treatment induced caspase-3 processing and Poly (ADP-Ribose) Polymerase 1 (PARP1) cleavage, indicating functional caspase-3 activation (Fig. 6c,d). Q-VD-Oph preincubation inhibited PARP1 cleavage as well as full processing of caspase-3 to p17 and p12 subunits for both imDNA co-transfection and ABT737 addition (Fig. 6c,d), confirming inhibition of caspase activation. While Q-VD-Oph restored puromycin incorporation during ABT737 incubation, it did not fully rescue puromycin incorporation in 3P-dsRNA:imDNA co-transfected cells (Fig. 6a). Caspase activation thus seems to be partially but not uniquely involved in the translational shutdown observed during imDNA co-transfection. In order to examine classical pathways leading to translational shutdown, we also probed the samples for eIF2α phosphorylation, a marker of the activation of the integrated stress response, and S6 dephosphorylation, as an indicator of mTOR pathway inhibition. Here, pI:C transfection led to increased eIF2α phosphorylation, as expected, since this nucleic acid is known to activate this pathway via Protein Kinase RNA-Activated (PKR)^49^. However, this could not be observed for 3P-dsRNA:imDNA co-transfection (Fig. 6e,f). Also, the mTOR inhibitor Rapamycin but not 3P-dsRNA:imDNA co-transfection suppressed S6 phosphorylation (Fig. 6g). Therefore, we excluded these classical translational control pathways as origin for the imDNA-induced translational shutdown.

Since Q-VD-Oph pre-incubation did not fully restore puromycin incorporation, we tested the impact of apoptotic caspase activation and inhibition on IFNB1 transcript levels. Interestingly, the direct mitochondrial apoptosis inducer ABT737 decreased IFNB1 mRNA transcript levels, which was increased again upon preincubation with Q-VD-Oph (Fig. 6j). These data match earlier studies indicating suppression of interferon transcription by apoptotic caspases activated by ABT737^32^,^33^. Furthermore, our data indicated that IFNB1 secretion was suppressed both at transcriptional as well as at translational levels.

By contrast, in 3P-dsRNA:imDNA co-transfected cells IFNB1 transcript levels were increased rather than reduced when compared to 3P-dsRNA transfected cells (Fig. 6j), as observed earlier (Fig. 3a). Thus, imDNA leads to both a mild increase of IFNB1 transcription levels as well as the complete suppression of its translation. However, inhibiting apoptotic caspases via Q-VD-Oph leads to a further, profound increase in IFNB1 mRNA levels, allowing for a transcriptional rescue of IFN-β secretion from this translational repression (Fig. 6j). Therefore, imDNA acts in an immunostimulatory manner by increasing IFNB1-transcription, yet this activity is masked by its simultaneous immunosuppressive action preventing IFN-β translation. Interestingly, in the same experiments, neither CXCL10 transcription nor secretion were significantly altered by the modulators imDNA and ABT737 nor by the pan-caspase inhibitor Q-VD-Oph (Fig. 6k).

To test whether Q-VD-Oph has a similar effect in primary cells, we repeated the experiment with primary monocytes. Here, Q-VD-Oph could induce a mild rescue of IFN-β secretion during imDNA co-transfection-mediated suppression (Supplementary Fig. S5a), confirming a role for caspases in this system as well. IFN-α secretion was also slightly increased (Supplementary Fig. S5b), and, in line with our data from THP-1, CXCL10 secretion was not significantly affected (Supplementary Fig. S5c). Treatment with ABT737 reduced IFN-α and IFN-β release, although it had a much more pronounced effect on IFN-β secretion. In addition, treatment with both ABT737 and Q-VD-Oph boosted secretion of both type-I-IFNs (Supplementary Fig. S5a,b). In contrast, at the transcriptional level, imDNA or ABT737 alone had little effect on IFNB1 mRNA levels, while the mRNA levels detected by IFNA2 and pan-IFN-α primers were only mildly reduced^50^ (Supplementary Fig. S5d,e,g). As was observed in THP-1, addition of Q-VD-Oph, profoundly increased IFNB1 mRNA levels in imDNA-treated cells. In addition, IFNA2 and pan-IFN-α levels were increased. Thus, monocytes recapitulate the phenotype we have observed in THP-1, in which Q-VD-Oph addition allows the transcriptional rescue of posttranslational blockage by imDNA, although this effect is somewhat more pronounced for IFN-β release than for IFN-α, which cannot be measured in THP-1. Again, as in THP-1, CXCL10 transcript levels were not significantly altered by any treatment (Supplemenary Fig. S5f).

Altogether, we conclude that caspase activation during imDNA transfection does not cause the observed global translational shutdown. Instead, caspase activation appears to suppress super-induction of IFN-β transcript levels during imDNA co-transfection, a process which can be blocked by the addition of Q-VD-Oph, leading to strongly increased IFN-β transcript levels. Interestingly, for IFN-α, only a mild rescue of transcript levels after caspase inhibition could be observed, and thus, the translational suppression induced by imDNA dominates the modulation of secretion. Nonetheless, neither imDNA, nor ABT737, nor caspase inhibition seem to affect the transcription or secretion of CXCL10, which was maintained at comparable levels under all of the type-I-IFN modulating conditions tested.

## Discussion

Here, we describe a novel form of post-transcriptional modulation of a type-I-IFN-dominated innate immune response. CpG ODN2216-like immune modulatory DNA (imDNA), when delivered into the cytosol, induced apoptosis and a global translational shutdown concomitant with reduced stimulatory nucleic acid-induced type-I-IFN secretion in monocytic cells. Essential for this inhibitory activity were the G-rich ends but not the CpG motifs present in ODN2216. In contrast to type-I-IFN secretion, the release of CXCL10 as well as several other chemokines was maintained under the influence of imDNA co-transfection. Furthermore, we demonstrate that CXCL10 secretion is cell-intrinsically rescued during the global translational shutdown demonstrating an enhanced translation, as reflected by an increased presence of the CXCL10 transcript in the higher-order polysomal fraction. imDNA also induced caspase-3 activation and PARP-1 cleavage, and IFN-β secretion could be rescued by pan-caspase inhibition. Furthermore, this differential regulation of IFN-β and CXCL10 was also observed during direct induction of mitochondrial apoptosis via the bcl-2 inhibitor ABT737. However, caspase inhibition did not fully rescue the imDNA-induced translational shutdown. Rather, it increased 3P-RNA-induced IFNB1 transcript levels, thus overcoming translational inhibition. At the same time, CXCL10 transcription and translation were not significantly affected by caspase activation or inhibition. To our knowledge, this is the first report on differential post-transcriptional regulation of type-I-IFNs and associated chemokines such as CXCL10 during severe cellular stress.

The observation of a further increase of IFN-β transcript levels by caspase inhibition under caspase-3 activating conditions is in line with earlier studies that demonstrated that apoptotic caspases suppress type-I-IFN induction during apoptotic cell death^32^,^33^. However, we observed that this is the case not only during cell-intrinsic IFN-β induction, but also during cytosolic stimulation. Although we used relatively low doses of 3P-dsRNA that do not markedly induce apoptosis, mild caspase-3 activation and PARP1 cleavage could also be observed in solely 3P-dsRNA transfected cells, and suppression of this activation upon Q-VD-Oph treatment, led to a mild increase in IFNB1 transcript levels and secretion. Increasing the activation of apoptotic caspases by ABT737 led to a blockade of IFN-β secretion by a decrease both in global translation and IFNB1 transcription. Since the pan-caspase inhibitor Q-VD-Oph was able to restore puromycin incorporation as well as IFNB1 transcription, there seems to be a caspase-mediated blockade at both the transcriptional and translational level. During imDNA co-transfection, the mild although not statistically significant improvement in global translation as well as increased activation of IFNB1-transcription during caspase inhibition suggest that similar mechanisms may be involved. However, in contrast to ABT737, imDNA co-transfection increased rather than reduced IFNB1 transcript levels and translation was not completely restored by caspase inhibition indicating the involvement of still unknown IFNB1-enhancing pathways as well as a caspase-independent mechanism of global translational shutdown.

We hypothesize that during 3P-RNA:imDNA co-transfection, transcript levels are balanced between super-induction by unknown mechanisms and suppression by apoptotic caspases. If the caspases are inhibited by Q-VD-Oph, the balance is shifted towards super-induction to an extent that allows for secretion that is comparable to solely 3P-RNA transfected cells.

Although imDNA from various suppliers was comparably active in most cases, for a minority of imDNA batches the balance appeared to be shifted towards transcript level suppression (data not shown). Here, even type-I-IFN transcript levels were reduced so that translational regulation was less pronounced. These batches also induced a reduction in the final CXCL10 concentration, possibly due to a more rapid cell death.

The increase in IFNB1 transcript levels, 6 h post transfection, may be due to a range of possible reasons: A simple increase in transcription, differences in transcription dynamics or increased transcript stability. We cannot exclude any of these possibilities, however, activation of IFN-β transcription by pan-caspase inhibition during apoptosis has been described in other studies^32^,^33^, which makes direct superactivation of transcription the most likely explanation.

An earlier study reported reduced cell viability in HEK293T cells following DNA transfection^51^. Cytosolic DNA led to an AMP-activated protein kinase (AMPK) dependent stress response and inhibition of mTOR signalling potentially leading to translational shutdown via hypophosphorylation of 4E-binding proteins and the ribosomal protein S6^51^. However, in our study, S6 phosphorylation (demonstrating mTOR signalling) was not affected by imDNA co-transfection. Furthermore, inhibition of mTOR signalling by rapamycin inhibited neither type-I-IFN-secretion nor puromycin incorporation in THP-1. Another well-studied global translational control mechanism is phosphorylation of the α-subunit of the translation initiation factor eIF2. eIF2α phosphorylation inhibits the formation of a fully functional translation initiation complex by preventing eIF2B from exchanging GDP for GTP to successfully form the ternary complex composed of methionyl-tRNA, GTP and eIF2^5^. Indeed, eIF2α phosphorylation-dependent translational blockade via PKR is common during viral infection and is considered an integral part of the anti-viral defence mechanisms during a type-I-IFN response. During viral infection or pI:C transfection of mouse embryonic fibroblasts, IFN-β and IL6 escape translational blockade, which is dependent on the eIF2α phosphatase GADD34. However, GADD34 deficiency did not influence PKR-dependent global translational blockade, indicating an additional, protein-specific GADD34-dependent layer of translational regulation that might resemble CXCL10 escape of global translational shutdown during imDNA co-transfection^52^. However, while pI:C transfection led to robust eIF2α phosphorylation via PKR activation, this was not the case for imDNA co-transfection or ABT737 treatment. Therefore, classical translational control mechanisms could be excluded, and further studies remain to be conducted to clarify the origin of the translational shutdown observed.

To date, a large range of post-transcriptional regulation mechanisms have been described that may explain how the differential translation of CXCL10 and the type-I-IFNs is actually regulated. Most known post-transcriptional regulation mechanisms are associated with features within the UTRs of an mRNA transcript. Indeed, some influence of the annotated 5′ and 3′ UTRs of IFNB1 and CXCL10 could be observed when they flanked GLuc in a reporter plasmid during the regular type-I-IFN response induced by the reporter plasmid transfection itself. However, imDNA co-transfection with these reporter plasmids suppressed GLuc activity in the supernatant of all transfected cells, including the ones with CXCL10 UTRs. However, it is possible that the annotated reference sequence does not reflect the complete 5′UTR of the CXCL10 transcript. Furthermore, imDNA co-transfection suppressed translation of GLuc mRNA lacking any UTRs, which may result from the synergistic stress effects of imDNA:plasmid co-transfection of THP-1, which are proficient in the cGAS and AIM2 pathways. However, it is also possible that there are translational regulations associated with cofactor recruitment during splicing, since the CXCL10 gene contains introns, while IFNB1 gene does not and is in this way comparable to the cDNA reporter systems. In addition, regulation via the poly(A)-tail may play a role since it has been described as a regulatory mechanism for both CCL5 and IFNB1 mRNA^53^,^54^.

A further effect of imDNA transfection is the induction of cell death, most probably apoptosis, since we observed profound activation of caspase-3 upon imDNA transfection. As observed earlier, however, pan-caspase inhibition did not rescue the cells from cell death or translational blockage but rather increased the transcript levels of IFN-β, leading to the increase in IFN-β secretion^32^,^33^. It will be an interesting question for further studies, whether cell death mechanisms other than caspase activation cause the translational shutdown observed or whether cell death is in fact, simply the result of a precedent translational shutdown.

Although we cannot exclude an additional transcriptional regulation of some IFN-α subtypes in monocytes, the differential post-transcriptional regulation of type-I-IFN-associated cytokines adds a new layer of regulation to the type-I IFN response that has not yet been extensively explored. An important physiological role for the translational activation of chemokines such as CXCL10 during cell stress and apoptosis might be the attraction of immune cells in the absence of type-I-IFNs. Many viruses target the global translational machinery by inhibiting classical translation initiation to divert it into the production of viral proteins, e.g. via IRESs^55^. Furthermore, if apoptotic caspases are activated during viral infection, this might suppress type-I-IFN secretion as well and chemokine secretion might be a way to still let immune cells patrol the infection tissue. It is attractive to hypothesize that innate immune cells have found a way to circumvent translational shutdown of chemokines and other cytokines to enable further immune cell recruitment.

Finally, a common marker of interferon-driven autoinflammatory diseases is CXCL10 in patient samples^56^. Type-I-IFNs are believed to be secreted at lower levels and more efficiently removed from the body fluids and thus harder to detect. However, our data indicates that CXCL10 and other type-I-IFN-associated chemokines can be secreted while type-I-IFN production itself is suppressed under certain cellular stress conditions. It is possible that one reason for the heterogeneous nature of interferonopathies is due to such effects and that, furthermore, the pathologies of some of these patients may not be driven by interferons themselves but rather by associated chemokines and cytokines.

In conclusion, our data indicates that the production of type-I-IFNs is not only tightly regulated in the transcriptional level but can also be inhibited post-transcriptionally during severe cell stress as induced by imDNA. At the same time, we provide evidence for mechanisms that ensure the unaffected secretion of CXCL10 and other chemokines during this stress response. From these observations, questions arise: What is the origin of the imDNA-induced stress response, which are the pathways involved? How is the differential translation of CXCL10 and type-I-IFN regulated? Given that an innate immune response does not occur in isolation and is often accompanied by cellular stress, the implications of this crosstalk may influence the outcome of an infection just as profoundly as the innate immune response itself.

## Methods

### Ethics statement

The studies of human PBMCs were approved by the local ethics committee (Ethikkommission der Medizinischen Fakultät Bonn) according to guidelines of the International Conference on Harmonisation of Technical Requirements for Registration of Pharmaceuticals for Human Use and Good Clinical Practice. Written informed consent was provided by voluntary blood donors.

### Cell culture and stimulation

Peripheral blood mononuclear cells (PBMC) were isolated from buffy coats from healthy blood donors and seeded as described previously (Herzner *et al*.^34^). Chloroquine (2.5 μg/ml) was added at least 30 min before transfection. Primary monocytes were isolated by anti-CD14-magnetic bead separation (MACS, Miltenyi Biotec) and seeded 2 × 10^5^/96-well in 200 μl/96-well. THP-1 were cultured in RPMI +10% FCS and 1 × P/S (Gibco) and seeded 6 × 10^4^/96-well in 100 μl medium. For stimulations, G-rich ODN and control ODN were heated to 85 °C for 5 min and immediately cooled to 4 °C or placed on ice until transfection. Per 96-well, 0,1 μg ODN was prediluted in 12,5 μl Opti-MEM and incubated for 15 min, then mixed 0,1 μg or 20 ng stimulatory nucleic acid in 12,5 μl Opti-MEM. Stimulatory DNA and 9.2 s RNA was applied at 0.1 μg/well, for PBMC, monocytes, and gradient centrifugation, 0,1 μg/96-well of a mixture of the inert RNA oligomer (CA)_10_ and IVT4 (127:1) was applied, all other experiments were conducted with 20 ng of IVT4^17^. Then, 0,5 μl Lipofectamine 2000 in 25 μl Opti-MEM was added, thoroughly mixed and incubated for 20 min. After further thorough mixing, 50 μl of transfection mix was added per 96-well. Supernatants were harvested 20 h after transfection and stored at −20 °C until detection of cytokine concentrations. To generate somatic knockout THP-1 cell lines, cells were electroporated with EF1-Cas9-U6- sgRNA expression plasmids targeting AIM2 (GATACTCTTGCTAACAGGCC(TGG)), cGAS (GGCCGCCCGTCCGCGCAACT(GGG)) and STING (CTAGCCCCCAAAGGGTCACC(AGG)), respectively. Successfully targeted single cell clones clones were identified by Sanger sequencing, immunoblot and functional testing.

### Detection of cytokine concentrations

IFN-α and CXCL10 concentrations were determined by Human IFN-α Matched Antibody Pairs (eBioscience) and Human CXCL10 ELISA Set (BD Biosciences). Human IFN-β was detected with Human IFN-β Module Set (Antigenix America) according to the manufacturer’s instruction, with some modifications. Briefly, ELISA plates were coated with capture antibody in PBS at 4 °C over night, blocked with assay buffer (1% PBS in PBS) for 1 h, washed 3× with wash buffer (PBS, 0,5% Tween 20), samples diluted in assay buffer, incubated for 2 h, plates washed 5×, incubated with detection antibody in assay buffer, washed 5×, incubated with Streptavidin-HRP (BD Biosciences) for 30 min, washed 7× and HRP activity detected with BD ELISA detection substrate. 64-plex multi-cytokine analysis was performed by EveTechnologies (Calgary, Alberta, Canada). Type-I-IFN activity was detected using HEK-blue IFN alpha/beta cells (Invivogen) according to the manufacturer’s protocol. SEAP activity was detected with the pNPP substrate.

### MTT assay

20 h after transfection, cells were incubated with 100 μl 0.5 mg/ml 3-(4,5-dimethylthiazol-2-yl)-2, 5-diphenyltetrazolium bromide (MTT) in medium for 1 h in the tissue culture incubator at 37 °C. 100 μl 10% SDS were added to stop the reaction and crystals dissolved by incubation over night at 37 °C. Substrate metabolization was detected by absorbance at 570 nm.

### RNA analysis

RNA from monocytes was isolated as described previously^34^, with the following modifications: RNA was DNaseI-digested after elution from columns, directly reverse transcribed using Superscript VILO reverse transcription Kit and cDNA quantified with LightCycler^®^ 480 Probes Master (Roche Applied Science). RNA from THP-1 was isolated and analysed as described previously^34^, however, DNA was removed by digestion with DNase I (Thermo) in solution after elution. Before cDNA synthesis, RNA was precipitated with standard ethanol precipitation: 0, 1 volume 3 M sodium acetate and 20 μg RNase-free glycogen were added, the solution carefully mixed. Then, 3 volumes ethanol were added and, after additional mixing, RNA was precipitated at 16000 rcf and RT for 15 minutes. The precipitate was washed with 70% Ethanol and solved in RNase-free water. RNA derived from sucrose gradient centrifugation experiments was spiked with *in vitro* generated GLuc RNA (1 ng/ml) and purified by dual extraction with water-saturated phenol (pH4, 5–5; Roth), followed by two consecutive extractions with chloroform:isoamylalcohol (24:1; Roth). The RNA was precipitated as described above, however the precipitation centrifugation step was altered to 16000 rcf and 4 °C for 30 minutes. RNA was reverse transcribed and analyzed as described previously^34^. Primers were: IFNA2 (Probe; TCCTGCTTGAAGGACAGACA; rev: TTTCAGCCTTTTGGAACTGG; probe #63 (Roche)); CXCL10 (probe; GAATGCTCTTTACTTCATGGACTTC; rev: GGTAGCCACTGAAAGAATTTGG; probe #88 (Roche)); β-actin (probe; fwd: GCACCCAGCACAATGAAGA; rev: CGATCCACACGGAGTACTTG; probe #63); IFNB1 (SYBR; fwd: CATTACCTGAAGGCCAAGGA; rev: CAGCATCTGCTGGTTGAAGA); CXCL10 (SYBR; fwd: TCTGAATCCAGAATCGAAGG; rev: CTCTGTGTGGTCCATCCTTG); CCL4 (SYBR; fwd: CTTCCTCGCAACTTTGTGGT; rev: GGATTCACTGGGATCAGCAC); CCL24 (SYBR; fwd: TGAGAACCGAGTGGTCAGC; rev; TCTGGACCCACTCCTGCTTG); CCL2 (SYBR; fwd: GCCTCCAGCATGAAAGTCTC; rev: AGGTGACTGGGGCATTGAT); CXCL8 (SYBR; fwd: CGGAAGGAACCATCTCACTG; rev: AGCACTCCTTGGCAAAACTG); CCL8 (SYBR; fwd: AGATGAAGGTTTCTGCAGCG; rev: AAAGCAGCAGGTGATTGGAA); TNF-α (SYBR; fwd: CTGCTGCACTTTGGAGTGAT; rev: AGATGATCTGACTGCCTGGG); CCL3 (SYBR; fwd: GGCTCTCTGCAACCAGTTCT; rev: TGAAATTCTGTGGAATCTGCC); GAPDH (SYBR; fwd: AAGGTGAAGGTCGGAGTCAA; rev: AATGAAGGGGTCATTGATGG); GLuc (fwd: GGTGCTCAAAGAGATGGAAGC; rev: CTTCTTCATCTTGGGCGTGC); pan-IFN-α (fwd: GTGAGGAAATACTTCCAAAGAATCAC; rev: TCTCATGATTTCTGCTCTGACAA)^50^.

### Sucrose gradient centrifugation

For the establishment of 10–50% sucrose gradient, 50%, 40%, 30%, 20% and 10% sucrose solution (10–50% sucrose; 10 mM Tris-HCl pH 8.0; 5 mM MgCl2; 100 mM KCl; mM DTT; 100 μg/ml cycloheximide) were overlayered stepwise (2 ml each) in a 15 ml ultracentrifugation tube, starting with 50%, ensuring complete freezing at −80 °C or on dry ice before adding the layer with the next lower sucrose concentration. Tubes were stored at −80 °C. 16 h before centrifugation, tubes were transferred to 4 °C to establish a uniform gradient. 8 h after transfection, cycloheximide (0, 1 mg/ml) was added to THP-1, the cells precipitated and lysed with RNA-lysis buffer (10 mM Tris-HCl; 5 mM MgCL2; 100 mM KCl, 2 mM DTT; 100 μg/ml cycloheximide; 1× cOmplete protease inhibitor (Roche Applied Science); 1% Triton X-100) at 4 °C for 30 min under gentle pivoting. Nuclei were precipitated (centrifugation at 1300 rcf, 8 min, 4 °C) and the supernatant layered over the established sucrose gradient. Ribonucleic complexes were separated by ultracentrifugation (35000 rcf, 100 min, 4 °C; Rotor SW 41 Ti). The gradient was led through an FPLC UV-detector (254 nm; ÄKTApurifier; GE Healthcare) by displacement with a 60% sucrose solution from the bottom of the ultracentrifugation tube. Afterwards, the gradient solutions were collected for quantitative RNA analysis.

### Immunoblot analysis

Cells were stimulated as described above. Cells precipitated by centrifugation (5 min, 4 °C, 350 rcf), resuspended in ice-cold PBS, again precipitated (400 rcf, 4 °C, 5 min) and lysed in Lämmli buffer (60 mM Tris pH6, 8; 2% SDS; 100 mM DTT, 5% glycerol; orange G). For the Sunset assay^48^, cells were incubated 10 min with 1 μg/ml puromycin prior to centrifugation. The samples were treated with ultrasound, denatured and reduced at 95 °C for 5 min and subjected to standard SDS-PAGE. The following antibodies were used for immunoblotting: Phospho-Chk2 (Thr68) (C13C1) Rabbit mAb; Phospho-Chk1 (Ser345) (133D3) Rabbit mAb; Phospho-Histone H2A.X (Ser139) (20E3) Rabbit mAb; PARP Antibody #9542; Caspase-3 Antibody #9662; Phospho-eIF2α (Ser51) (D9G8) XP^®^ Rabbit mAb; eIF2α Antibody #9722; Phospho-S6 Ribosomal Protein (Ser235/236) (D57.2.2E) XP^®^ Rabbit mAb (all Cell Signalling Technologies), Puromycin (12D10) mouse mAB (Merck Millipore), β-Actin Antibody (C4) HRP (Santa Cruz).

### Statistical analysis

If not stated otherwise, statistical analysis was performed by repeated-measures two-sided, one-way ANOVA with data matched according to donor (PBMCs) or the same experiment (cell lines) with GraphPad Prism 6. If the P-value calculated by ANOVA was considered significant (<0.05), individual comparisons were performed with Fisher’s Least Significant Difference post-hoc test (if not stated otherwise).

## Additional Information

**How to cite this article**: Herzner, A.-M. *et al*. G-rich DNA-induced stress response blocks type-I-IFN but not CXCL10 secretion in monocytes. *Sci. Rep.* **6**, 38405; doi: 10.1038/srep38405 (2016).

**Publisher's note:** Springer Nature remains neutral with regard to jurisdictional claims in published maps and institutional affiliations.

## Supplementary Material

Supplementary Information

## Figures and Tables

**Table 1:** Nucleotide sequences of modulatory DNAs/RNA.

ODN 2216	GGGGGACGATCGTCGGGGGG
ODN 2216 (G > T)	TTTTTACGATCGTCGTTTTT
imDNA	GGGGGGCAGCATGCTGGGGG
imDNA-T	TTTTTTCAGCATGCTGTTTT
imDNA-A	AAAAAACAGCATGCTGAAAA
imDNA-C	CCCCCCCAGCATGCTGCCCC
cimDNA	GGGGGGCAGCTTGCTGGGGG
R2023 (RNA)	GGGGGACGUACGUCGGGGGG
