# Supplementary material for: G-rich DNA-induced stress response blocks type-I-IFN but not CXCL10 secretion in monocytes
Source: Sci Rep. 2016 Dec 12;6:38405. doi: 10.1038/srep38405 (PMC5150577; doi:10.1038/srep38405)
Supplement: Supplementary Information [file srep38405-s1.pdf]

Supplementary Data

G-rich DNA-induced stress response blocks type-I-IFN but not CXCL10 secretion in monocytes

Anna-Maria Herzner[1,3]*, Steven Wolter[1], Thomas Zillinger[1,2], Saskia Schmitz[1], Winfried Barchet[1,2], Gunther Hartmann[1], Eva Bartok[1], Martin Schlee[1]

Affiliations:
[1]Institute of Clinical Chemistry and Clinical Pharmacology, University Hospital Bonn, Bonn, Germany.
[2]German Center of Infectious Disease, Cologne-Bonn, Germany.
[3]Current address: Department of Human Genetics, Genentech, Inc., South San Francisco, CA 94080, USA

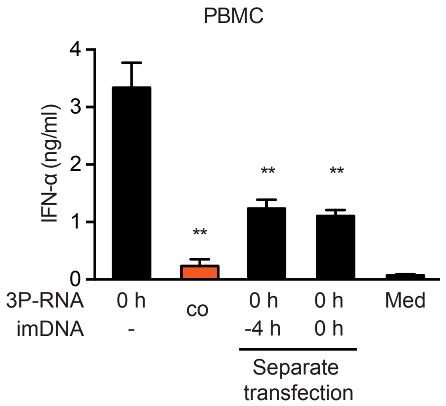

Supplementary Figure S1: Separated transfection of 3PRNA and imDNA
IFN-α concentration in supernatants, 20 h after transfection of PBMC with 3P-RNA (set as 0 h). 3P-RNA was either transfected separately (0 h) or together with imDNA (co). imDNA was transfected either 4 h before 3P-RNA (-4 h) or simultaniously (0 h). - : no imDNA transfection, Med: Medium. Data are pooled from two experiments with two biological replicates in each experiment. Data represent mean+s.e.m. (n=4). **P ≤ 0.01 (repeated measures one-way ANOVA followed by Fisher's LSD post-hoc test).

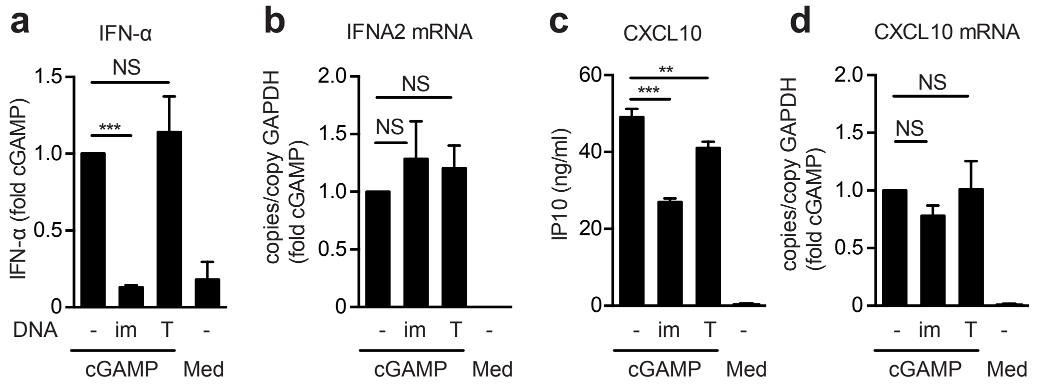

Supplementary Figure S2: cGAMP-induced IFNa secretion is blocked on the post-transcriptional level
Primary CD14+ Monocytes were incubated with 2'3'cGAMP and simultatiously transfected with imDNA (im) or cimDNA (cim). (**a**,**c**) IFN-α (**a**) or CXCL10 (**c**) concentration in supernatants, 20 h after stimulation with cGAMP and/or transfection with imDNA (im) or imDNA-T (T). (**b**,**d**) mRNA transcript levels 6 h post stimulation of IFNA2 (**b**) or CXCL10 (**d**), displayed as relative to transcript levels of cell stimulated with cGAMP. - : no DNA transfection, Med: Medium. Data are pooled from two experiments with two biological replicates in each experiment. Data represent mean+s.e.m. (n=4). NS, not sigificant (P>0.05); **P ≤ 0.01; ***P≤0.001 (repeated measures one-way ANOVA followed by Fisher's LSD post-hoc test).

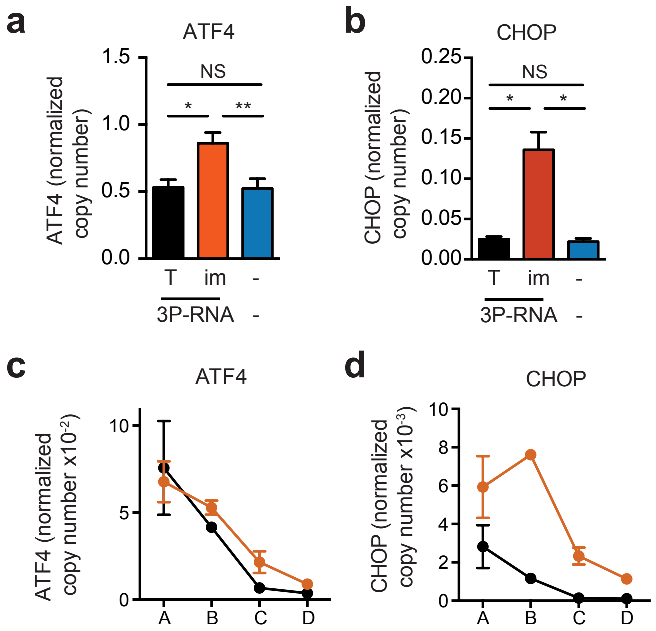

Supplementary Figure S3: Induction of cellular stress response markers
(**a,b**) ATF4 (**a**) or CHOP (**b**) mRNA expression of THP-1, untreated (blue; “-”) or transfected with 3P-RNA (3P-RNA) and imDNA-T (black, “T”) or imDNA (vermilion, “im”), 8 h post transfection. Data are normalized to GAPDH mRNA expression. (**c,d**) ATF4 (**c**) and CHOP (**d**) mRNA, detected in the respective fractions after ribonucleoparticle separation as in Fig. 3D/E, of THP-1, transfected with 3P-RNA and imDNA-T (black) or imDNA, respectively (vermilion). Data are normalized to spiked-in GLuc mRNA. Data are pooled from three (**a,b**) biological replicates (mean and s.e.m.) or representative of three independent experiments (**c,d**) and displayed as technical duplicates (mean and s.d.). (**a,b**) NS, not significant ($P > 0.05$); $^{*}P \leq 0.05$; $^{**}P \leq 0.01$ (repeated measures one-way ANOVA followed by Fisher’s LSD post-hoc test). The following primers were used for SYBR-based qPCR: ATF4: fwd: GGGA-CAGATTGGATGTTGGAGA; rev: ACCCAACAGGGCATCCAAGT; CHOP: fwd: CAGACT-GATCCAACTGCAG; rev: GACTGGAATCTGGAGAGTG

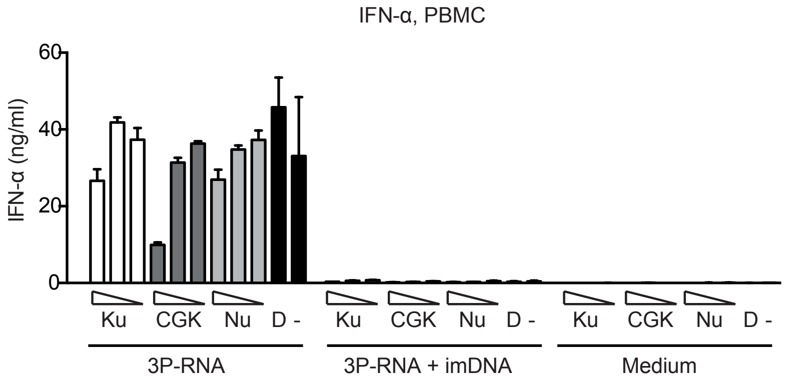

Supplementary Figure S4: DNA damage inhibitors do not restore IFN-α secretion
IFN-α concentrations in the supernatants of PBMC, treated with chloroquine (2,5 μg/ml) and DNA damage inhibitors Ku55933 (110 nm; 22 nm; 7.3 nm), CGK733 (6 μM; 3 μM; 1 M) or Nu7062 (11.5 μM; 2.3 μM; 0.77 μM) transfected with indicated nucleic acids. D,DMSO control. IFN-α concentrations were detected 20 h post transfection. Data are pooled from two donors (mean and s.e.m).

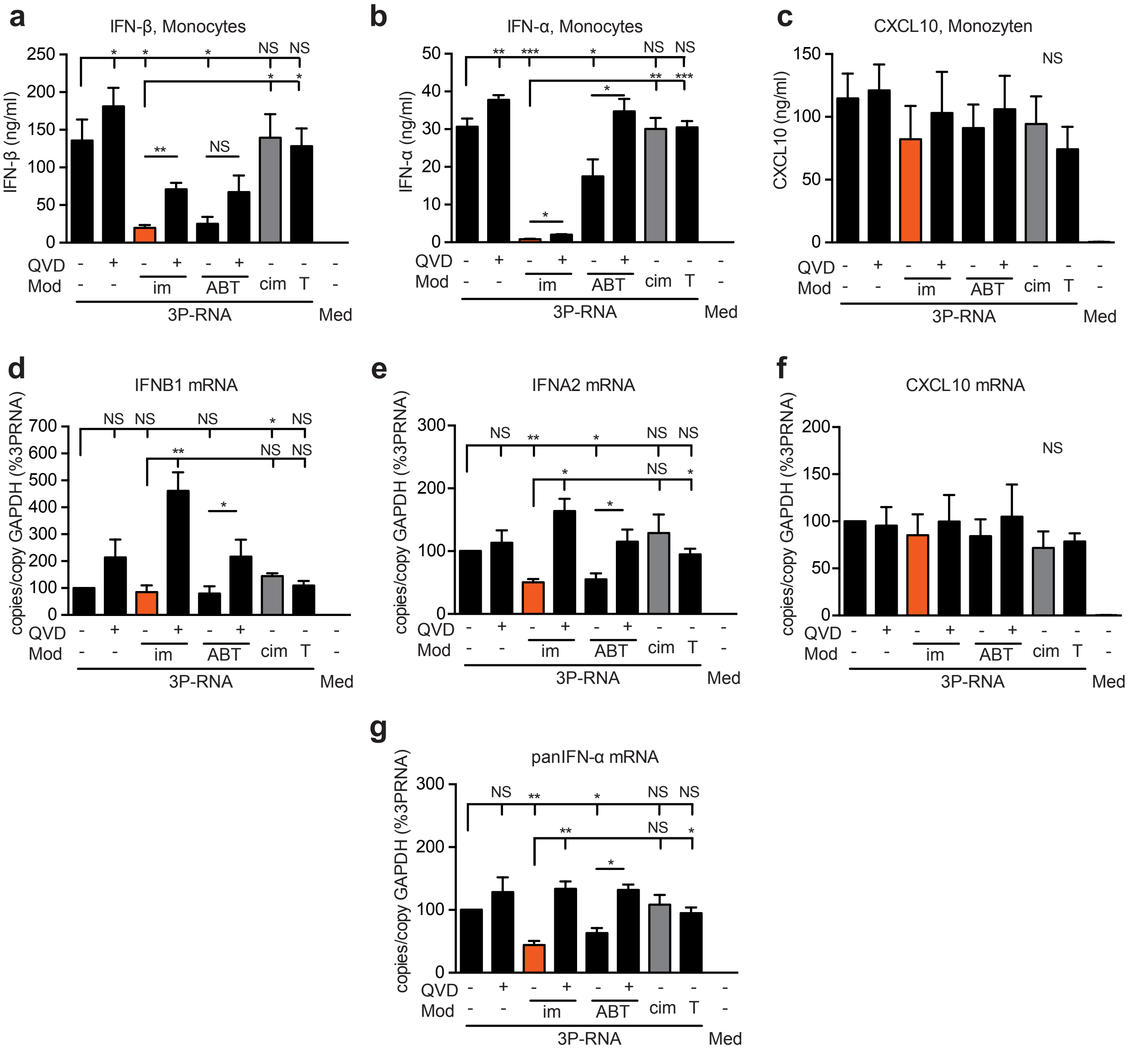

Supplementary Figure S5: Q-VD-Oph modulates type-I-IFN secretion in primary monocytes
(**a-c**) IFN-β (**a**), IFN-α (**a**) or CXCL10 concentrations in the supernatants of monocytes, pretreated with Q-VD-Oph (QVD), transfected with 3P-RNA and/or imDNA (im), cimDNA (cim) or imDNA-T (T) and/or treated with ABT737 (ABT), measured 20 h after transfection. (**d-g**): Expression of IFNB1 (**d**), IFNA2 (**e**) or CXCL10 (**f**) mRNA or IFN-α mRNA detected by pan IFN-α Primers (**g**), normalized to GAPDH mRNA, measured by quantitative RT-PCR 6h post transfection. (**d-g**) Results are displayed as relative to those measured for 3P-RNA transfected cells, set as 100%. Mod, modulatory agent. NS, not significant ($P > 0.05$); *$P \leq 0.05$; **$P \leq 0.01$; ***$P \leq 0.001$ (repeated measures one-way ANOVA followed by Fisher‘s LSD post-hoc test). Data are pooled from two experiments with two biological replicates in each experiment (mean and s.e.m. of n = 4 donors).
